# Gut microbiome composition, not alpha diversity, is associated with survival in a natural vertebrate population

**DOI:** 10.1186/s42523-021-00149-6

**Published:** 2021-12-20

**Authors:** Sarah F. Worsley, Charli S. Davies, Maria-Elena Mannarelli, Matthew I. Hutchings, Jan Komdeur, Terry Burke, Hannah L. Dugdale, David S. Richardson

**Affiliations:** 1 School of Biological Sciences, University of East Anglia, Norwich Research Park, Norfolk, NR4 7TJ UK; 2 Department of Molecular Microbiology, John Innes Centre, Norwich Research Park, Norwich, NR4 7UH UK; 3 Groningen Institute for Evolutionary Life Sciences (GELIFES), University of Groningen, P.O. Box 11103, 9700 CC Groningen, The Netherlands; 4 Department of Animal and Plant Sciences, NERC Biomolecular Analysis Facility, University of Sheffield, Sheffield, S10 2TN UK; 5 Faculty of Biological Sciences, School of Biology, University of Leeds, Leeds, LS2 9JT UK; 6 Nature Seychelles, Roche Caiman, Mahé Republic of Seychelles

**Keywords:** Gut microbiome, Microbial diversity, Fitness, Life history, *Acrocephalus sechellensis*

## Abstract

**Background:**

The vertebrate gut microbiome (GM) can vary substantially across individuals within the same natural population. Although there is evidence linking the GM to health in captive animals, very little is known about the consequences of GM variation for host fitness in the wild. Here, we explore the relationship between faecal microbiome diversity, body condition, and survival using data from the long-term study of a discrete natural population of the Seychelles warbler (*Acrocephalus sechellensis*) on Cousin Island. To our knowledge, this is the first time that GM differences associated with survival have been fully characterised for a natural vertebrate species, across multiple age groups and breeding seasons.

**Results:**

We identified substantial variation in GM community structure among sampled individuals, which was partially explained by breeding season (5% of the variance), and host age class (up to 1% of the variance). We also identified significant differences in GM community membership between adult birds that survived, versus those that had died by the following breeding season. Individuals that died carried increased abundances of taxa that are known to be opportunistic pathogens, including several ASVs in the genus *Mycobacterium*. However, there was no association between GM alpha diversity (the diversity of bacterial taxa within a sample) and survival to the next breeding season, or with individual body condition. Additionally, we found no association between GM community membership and individual body condition.

**Conclusions:**

These results demonstrate that components of the vertebrate GM can be associated with host fitness in the wild. However, further research is needed to establish whether changes in bacterial abundance contribute to, or are only correlated with, differential survival; this will add to our understanding of the importance of the GM in the evolution of host species living in natural populations.

**Supplementary Information:**

The online version contains supplementary material available at 10.1186/s42523-021-00149-6.

## Background

Almost all eukaryotic organisms accumulate diverse communities of microorganisms that, over evolutionary time, have become an integral part of the host’s ecology and biological function [1]. In vertebrates, the gut microbiome (GM) consists of a complex community of microbes, including bacteria, archaea, viruses, and microbial eukaryotes, which can play an important role in host processes such as digestion, behaviour, development, and immunity [2–5]. For example, beneficial members of the GM facilitate the metabolism of otherwise indigestible dietary components and, in doing so, release a range of essential nutrients including short-chain fatty acids; these metabolites can make substantial contributions to the caloric requirements of animal species [6]. They also play a significant role in immune cell development, as well as cognition via the gut-brain axis [4, 6]. As such, experimental studies have shown that GM disruption can have a significant impact upon the health and survival of a wide range of host organisms in captivity [2, 7–9]. For example, a reduction in GM diversity, or an imbalance in GM composition, has often been linked to poor host health and the onset of disease in humans and captive animals [9–11].

Captive organisms harbour very different, often depauperate, microbial communities relative to individuals living in natural populations [7, 12, 13]. Indeed, the rewilding of captive mice (*Mus musculus*) causes a rapid shift in the composition and complexity of the GM which, in turn, alters the host’s immune system and susceptibility to disease [14, 15]. Wild animals are exposed to highly complex and dynamic environmental pressures which are often poorly represented in captive systems, but that could interact with, or override, the impact of the GM on the host [16]. Furthermore, high levels of inbreeding in captive animal lines results in host genetic homogeneity, which can artificially reduce GM diversity in these populations relative to those in the wild [16, 17]. As a result, it is unclear whether the relationships between GM variation and host health that are observed in captivity are representative of those that arise in wild populations. Additionally, whether GM variation is linked to host fitness components, such as survival, in wild populations is largely unknown, meaning that we have a relatively poor understanding of the evolutionary significance of the GM.

The emergence of high-throughput sequencing technologies, in combination with the development of effective, non-invasive sampling techniques, has resulted in a recent proliferation of studies investigating the GM of wild animals. Several studies have demonstrated interspecific differences in GM characteristics [18–20], and also variation between groups [21–23] or individuals [24–26] within the same natural population. Most of these studies have focused on investigating the drivers of intraspecific variation in the bacterial component of the GM, identifying a suite of environmental factors that can influence this, including habitat quality and dietary differences [25–27], as well as host-related traits, such as age, sex, and host genotype [24, 28, 29]. However, very few studies have investigated the consequences of individual GM variation for host health and fitness (survival and/or reproductive success) in natural populations.

A small number of studies have explored interactions between GM composition and host infection in the wild [26, 30, 31], however, there is conflicting evidence over whether, and how, GM diversity influences host condition. For example, one study on great tits (*Parus major*) demonstrated a positive relationship between GM species richness and nestling body mass [32], while other studies on the same (and other) species have shown the opposite effect, suggesting that, although the GM can contribute to host nutrition and immunity, there could be costs to maintaining a diverse microbiome in young birds [33, 34]. The relationship between particular component taxa in the GM and host body condition is complex and has been shown to vary across host species [33]. For example, increased abundances of certain *Lactobacillus* species were associated with nestling weight gain in wild great tits [34]; other species in this genus have also been associated with weight modification and altered feed conversion rates in captive animal species [35]. Similarly, abundances of the bacterial genera *Ruminiclostridium* and *Rikenella* have been linked to increased body mass in wild house mice, although this relationship was shown to vary depending on the study site [36]. In contrast, no correlation was identified between the abundance of specific bacterial taxa and growth in nestling house sparrows (*Passer domesticus*) [33].

We are only aware of two studies that have investigated the link between GM characteristics and survival in wild vertebrate species. In great tit nestlings, a negative, time-lagged association between GM alpha diversity and body mass was identified; while, separately in the same study, reduced body mass was associated with a lower probability of successful fledging [34]. Similarly, in a study on adult blue tits (*Cyanistes caeruleus*), reduced species richness and the presence of pathogenic *Campylobacter* species in the GM was associated with reduced annual survival, although gel electrophoresis was used to assess bacterial community structure in this instance, which limited resolution [37]. Further studies that investigate associations between the GM and fitness components across different life stages, and in other host species, are essential if we are to understand the evolutionary and ecological importance of the GM.

The main barrier to studying fitness in natural systems is the need for detailed longitudinal monitoring of individuals and accurate measures of fitness components. This requires accurate and complete parentage assignment and that measures of survival are not confounded with individual dispersal away from the study site. The Seychelles warbler (*Acrocephalus sechellensis*)—an insectivorous passerine endemic to the Seychelles Archipelago—provides an excellent model system for studying fitness in the wild. The entire warbler population on Cousin Island has been intensely monitored since 1985, with the majority of individuals colour ringed (> 96% since 1997), enabling comprehensive longitudinal monitoring of behaviour, annual fitness, and life-history parameters [38–40]. The population is closed, with virtually no inter-island movement, meaning that accurate measures of survival can be achieved [41]. Seychelles warblers are long-lived for a small passerine, with a median life expectancy at fledging of 5.5 years and a maximum recorded lifespan of 19 years [42, 43]. As Seychelles warblers lack natural predators and experience limited climatic variation or human disturbances, extrinsic mortality is lower than in many other passerine species living in temperate regions [44]. Indeed, previous research on this system has shown that the average annual survival probability is exceptionally high in adults (0.84); this is also the case for juvenile age classes, although mortality is greater in the first year of life (an average annual survival probability of 0.61) [44].

Since 2017 a non-invasive method of sampling the Seychelles warbler GM, via the collection of faecal matter, has been routinely used [45]. Amplicon sequencing of the bacterial component of samples taken across three breeding seasons has previously demonstrated that GM diversity varies substantially across individuals within the Cousin population, and is associated with host immunogenetic variation, age, and seasonal differences [45]. Here, we use faecal samples taken across six consecutive breeding seasons (spanning four years) to investigate whether GM variation is linked to individual differences in condition and survival in the Seychelles warbler. First, we assess whether variation in GM alpha diversity and composition is associated with body condition. Bacterial alpha diversity may be positively associated with body condition, for example if greater species richness translates into increased functional capacity and resource availability for host growth and immunity [32]. Alternatively, high GM diversity may be costly to maintain [33] or, more rarely, be indicative of poor health and GM instability [46] and thus, may be negatively associated with body condition. Specific bacterial taxa may also enhance or reduce host condition, depending on the extent to which they are beneficial or pathogenic to the host [7]. Second, we test whether GM diversity is associated with differential survival across individuals. We hypothesise that high GM diversity, as a general cause and consequence of good health in captive systems [4, 10], will be associated with a greater probability of survival to the next breeding season. However, the opposite relationship could also occur, if there are costs associated with maintaining a diverse microbiome. We also expect GM composition to differ between individuals that survived compared to those that die by the next breeding season if this corresponds to altered levels of pathogenic or beneficial bacterial families.

## Methods

### Study species and sample collection

The study was carried out on the population of Seychelles warblers inhabiting Cousin Island (29 ha; 04° 20′ S, 55° 40′ E). This stable population consists of *ca* 320 adult individuals [47], nearly all of which (> 96%) have been ringed with a unique combination of a British Trust for Ornithology (BTO) metal ring and three plastic colour rings [48]. Seychelles warblers are long-lived, with a median life expectancy at fledging of 5.5 years and a maximum recorded lifespan of 19 years [42, 43]. Population monitoring takes place twice a year, during the major (June–September) and minor (January–March) breeding seasons, respectively [49]. As the annual resighting probability of adult individuals is very high (98% ± 1%) [50] and inter-island dispersal is virtually absent [41], individuals not seen during a breeding season can be confidently assumed to be dead. Faecal sampling took place in the major breeding periods of 2017–2019, and the minor breeding periods of 2018–2020. As it was not possible to carry out the population census for the major breeding season of 2020 (due to the Covid-19 pandemic), information on the survival of individuals sampled in the minor breeding period of 2020 is not known; as such, samples collected in 2020 are included in overall analyses of GM variation but not in survival analyses.

The population of warblers on Cousin Island is structured into *ca* 115 territories which are defended year-round [47]. Seychelles warblers are insectivorous and take the majority of their insect food from leaves; thus, for each territory, an index of territory quality was calculated based on the number of insect prey available, the territory size, and foliage cover present in that breeding season [51]. For territories with missing quality measures in a season, quality was calculated as the average from the preceding and following breeding periods [as in 44].

During each breeding season, individuals were caught using mist nets and birds were weighed (±  0.1 g) using a 50 g Pesola balance. Right tarsus length (±  0.1 mm) was measured using vernier callipers. A blood sample was taken via brachial venipuncture and DNA was extracted using the DNeasy Blood and Tissue kit (Qiagen, Crawley, UK) according to the manufacturer’s instructions. Molecular sexing was subsequently carried out using a PCR-based method [43, 52]. Each individual was classified into one of the following age classes based on a combination of hatch date, behavioural observations, and eye-colour, which changes from grey in fledglings to red-brown in adult individuals [51]: nestling (in the nest), fledgling (1–3 months), old fledgling (3–5 months, and less reliant on parents for food), sub-adults (5–12 months), or adults (> 12 months).

To sample the GM, captured birds were placed into a disposable, flat-bottomed paper bag containing a sterilised weigh boat protected by a metal grate; this set-up follows an established protocol [45, 53] and allows faecal matter to fall into the tray whilst minimising the possibility of contamination, for example, from the bird’s surface. Each bird was removed from the bag after defecation (or after 30 min). A sterile flocked swab was used to collect faecal samples into a sterile microcentrifuge tube containing 1 ml of absolute ethanol. During sample collection, control swabs were also taken from empty collection bags and from field worker’s hands to capture possible sources of contamination. All samples were stored at 4 °C for the remainder of the field season, before transferring to − 80 °C for long-term storage. Over the course of six consecutive sampling seasons, 546 faecal samples were collected from 326 individuals on Cousin Island.

### DNA extraction from faecal samples and sequencing

The DNeasy PowerSoil Kit (Qiagen) was used to extract total genomic DNA from all faecal and control samples, according to a modified version of the manufacturer’s instructions [see 45]. Samples were randomised across extractions. Two negative extraction controls (using blank sterile swabs) were also carried out per extraction kit. Positive controls were extracted from a D6300 Microbial Community Standard (ZymoBIOMICS) to enable extraction quality and the reproducibility of sequencing to be established. In addition, ten randomly selected faecal samples were extracted twice to assess the repeatability of the extraction method. A Qubit dsDNA High Sensitivity Assay kit (Invitrogen) was used to quantify DNA concentration and samples were submitted for 16S rRNA gene amplicon sequencing at the NEOF Centre for Genomic Research, Liverpool. In total, 638 samples were sent for sequencing; this included 27 control samples (11 collection controls, 10 negative extraction controls, and 6 positive controls) and 611 faecal samples which included 55 samples that were sequenced twice (either in the same run or across different runs) as well as 10 repeat extractions. The universal primers 515F and 806R [54], which amplify the V4 region of the 16S rRNA gene, were used to generate amplicon libraries [see 45 for details]; libraries underwent paired-end, 2 × 250 bp sequencing across four Illumina MiSeq runs. Samples collected in 2017 and 2018 were randomised across plates in the first three runs. Samples collected in 2019 and 2020 were randomised across plates in the fourth run. As noted above, 55 samples were sequenced again across runs to check for batch effects.

### Data processing

Sequences were imported into QIIME2 2019.10 [55] for processing. The DADA2 plugin [56] was used to truncate forward and reverse sequences at 240 base pairs, and trim 13 base pairs from the 5′ end of reads to remove low quality base calls. Amplicon Sequencing Variants (ASVs) were inferred for each sample, followed by dereplication, paired-end joining, and the removal of chimeras. Files from the four separate sequencing runs were then merged, resulting in a total of 35,905,397 reads (mean per sample = 56,278 ± 52,716 SD). A mid-point rooted phylogeny was constructed using MAFFT [57] and the Fast Tree approach [58]. ASVs were taxonomically classified by training a naïve-Bayes classifier on the SILVA reference database 132 for 16S sequences. Sequences classified as chloroplast or mitochondria were removed, leaving 34,480,836 reads in total. One negative control and one faecal sample contained no reads after this filtering step, leaving 636 samples in total. One ASV assigned to the genus *Delftia* was removed from all samples sequenced in the first run, as it made up ~ 90% of reads in the extraction control for this run but was largely absent from samples in other runs. Similarly, ASVs assigned to the genus *Limnobacter* and the family *Veillonellaceae* were also removed from samples, as these were abundant in the negative extraction controls of runs three and four, respectively. All singleton reads were also removed from the dataset, as these represent possible sequencing contaminants. Eight unique ASVs were identified in each of the positive control samples; these corresponded taxonomically to the eight bacterial isolates making up the microbial community standard. The final sample metadata, ASV and taxonomy tables were all exported from QIIME2 into R 4.0.2 [59] and were further processed using *phyloseq* 1.32.0 [60]. Before conducting downstream analysis, sequences were filtered to remove all non-bacterial sequences, as well as ASVs that were unassigned at phylum level. Control samples were removed prior to analysis. There were 55,664 ASVs in the remaining 610 faecal samples. Sample completeness and rarefaction curves were generated using the R package *iNEXT* 2.0.20, with 50 bootstrap replicates per sample [61]. Sample completeness plateaued at approximately 10,000 reads (Additional file 1: Fig. S1); therefore, all faecal samples with fewer than 10,000 reads were excluded from downstream analyses (23 samples).

### Statistical analyses

#### Alpha diversity

All samples that remained after filtering were rarefied to a depth of 10,000 reads before calculating alpha diversity metrics, leaving 49,116 ASVs across 587 samples. The metrics Chao1 richness (estimates the number of different bacterial ASVs in a sample) and Shannon diversity (the number of ASVs and the evenness of their abundances within a sample) were calculated using *phyloseq* 1.32.0 [60]. Faith’s phylogenetic diversity (PD) was calculated using *picante* 1.8.2 [62]. One sample was removed from all downstream analysis due to having an exceptionally small alpha diversity value; extraction notes suggested that this had been an unusually small sample (586 samples retained). To measure how consistent alpha diversity measurements were across different extractions and sequencing runs, pairwise Euclidean distances were calculated based on the Shannon diversity of samples that had been extracted twice, or instances where DNA from the same extraction had been sequenced twice across sequencing runs (10 and 55 samples, respectively). A bootstrapping approach was used to statistically compare how pairwise distances varied based on the type of sample comparison (i.e. duplicate extractions of the same sample, sequencing duplicates of the same extraction across runs, or between sample comparisons). To this end, the Kruskal–Wallis test statistic and *P* value were permuted, resampling (99,999 permutations) with stratification specified by sample ID, using *boot* 1.3.28 [63, 64]. This approach corrects for the non-independence of comparisons which include the same sample IDs. A bootstrapped post-hoc Dunn’s Multiple Comparisons test was then carried out in the same way, using *FSA* 0.8.32 [65], with Benjamini–Hochberg false discovery rate corrected *P* values [66].

Following this analysis, duplicate samples were filtered, such that only the sample with the highest read count was retained (leaving 527 samples). Where multiple samples had been taken from the same individual during the same catch, only a single sample was retained. Samples were prioritised if they had been taken from the sterile tray, followed by those from inside of the bag. If both samples were collected from the same location, then the sample with the highest read count was retained (470 samples were retained following filtering).

##### Body condition

Size-corrected body mass has been used as an indicator of body condition for several vertebrate species [67], including the Seychelles warbler [68, 69]. Thus, body mass was used as a proxy for condition in analyses. Individuals that did not have a corresponding body mass measurement taken at the time of sampling were removed from the dataset (leaving 447 samples). Nestlings were also removed due to a small sample size (12 samples), as well as individuals with a “floater” status that had no assigned territory (eight samples). Two female breeding individuals were also excluded as they were recorded as carrying eggs at the time of sampling which increased their body mass. A total of 425 samples from 296 individuals were retained in the analysis. A linear mixed effects model (LMM) was constructed using *lme4* 1.1.27 [70], with body mass as the response variable and right tarsus length as a covariate to account for structural size differences between individuals. GM alpha diversity and its squared value, sex, age class (fledgling, old fledgling, sub-adult, or adult), territory quality, and the sampling field period (Major 2017, Minor 2018, Major 2018, Minor 2019, Major 2019, Minor 2020) were included as fixed effects in the model. The time of sampling (minutes from sunrise at 06:00 am) was also included as a fixed effect, as it influences body mass in the Seychelles warbler [68]. Bird ID, territory ID, and observer ID were all included as random intercepts to control for the non-independence of samples. Continuous predictors were centred and scaled to a mean of 0 and standard deviation of 0.5, using *arm* 1.11.2 [71]. Separate models were run using Shannon diversity, Chao1 richness, or Faith’s PD as the measure of GM alpha diversity, to check if results were consistent across metrics. The resulting *P* values were corrected for multiple testing using the Benjamini–Hochberg false discovery rate correction [66]. Chao1 richness and Faith’s PD were both log-transformed in analyses to improve residual fit. Biologically relevant interactions were also included in the model but were removed sequentially (in order of least significance), followed by the squared terms, if they were not significant, to enable interpretation of the first-order effects. The R package *car* 3.0.10 [72] was used to calculate Variance Inflation Factors (VIFs); VIFs were < 3 for all terms in the model. The R package *DHARMA* 0.4.1 [73] was used to carry out model diagnostics. Marginal and conditional R^2^ values were calculated using the r.squaredGLMM function in the package *MuMIn* 1.43.17 [74].

##### Survival

The 470 samples were filtered to remove 72 samples taken in 2020 as these samples had no follow-up census in the next breeding season to assess bird survival. Where multiple samples were available from the same bird over different field periods, only the last sample was included, as there were too few individuals with multiple samples to control for pseudoreplication in the model (< 30% of individuals had multiple samples once 2020 samples had been removed). Samples from nestlings and floaters were also removed, as above. A final total of 264 samples/individuals were included in the analysis (226 individuals that survived, 38 individuals that died). Although the exact date of death is not known, for 25 of the individuals that died by the next breeding season, the point of GM sampling was the last time they were observed in the population. However, the remaining 13 individuals that died were observed (but not sampled) again in the same breeding season as GM sampling took place; in some cases, this was up to eleven weeks after their last GM sample was taken. There was also a median period of 4.5 months between the point when GM samples were taken, and when the population was next censused to assess survival. Thus, it is possible that some of the individuals were sampled several months before their point of death. A Generalised Linear Model (GLM) with a binomial error structure and logit link function was constructed using *stats* 4.0.2 [59]. Survival to the next breeding season was included as a binary response variable (0—died, 1—survived). GM alpha diversity and its squared term, bird age class (fledgling, old fledgling, sub-adult, or adult), sex, and territory quality were included as independent variables. Sampling year was also included, to control for differences in survival probabilities between years [44]. VIFs were < 3 for all model terms, continuous variables were centred and scaled, and interactions were removed sequentially if not significant to interpret the first-order effects.

#### Beta diversity analysis

The unrarefied reads (filtered to remove samples with < 10,000 reads) were used. Samples were processed to remove exceptionally rare taxa that could disproportionately influence beta diversity metrics. As such, ASVs were excluded if they had fewer than 50 reads in total across all samples and/or were present in less than 2% of samples. This retained 3,057 ASVs across 586 samples. ASV abundances were then transformed using the Centered Log Ratio (CLR) transform function in *microbiome* 1.12.0 [75]. The CLR transformation produces values that are scale invariant (i.e. not influenced by differences in library sizes across samples) and controls for the compositional nature of microbiome datasets [76]. The consistency of beta diversity estimates across different extractions and sequencing runs was assessed as described for Shannon diversity, but using pairwise Euclidean distances calculated using the CLR transformed ASV abundances.

To identify whether compositional differences in the GM were associated with host age class, sex, or environmental variables, sequencing and catch duplicates were first removed (as described above). Samples taken from nestlings and floaters were also removed (as described above), leaving 450 samples from 309 individuals. A Euclidean distance matrix was then calculated using the CLR-transformed ASV abundances. To quantify differences in beta diversity between groups of samples, a Permutational Analysis of Variance (PERMANOVA) was performed using the *adonis2* function within the R package *vegan* 2.5.7 [77, 78], with 9999 permutations. Individual age class (fledgling, old fledgling, sub-adult, adult) and sex were included as predictors, as well as territory quality (as a proxy for dietary differences across the island and local competition for food) and the corresponding sampling field period (Major 2017, Minor 2018, Major 2018, Minor 2019, Major 2019, Minor 2020). Bird ID was included as a blocking factor to control for repeated sampling. The function *betadisper* was used to check for the homogeneity of group dispersion values [77, 78]. Pairwise PERMANOVA analyses were conducted using pairwiseAdonis 0.0.1 [79]—the Benjamini and Hochberg method [66] was used to correct *P* values for multiple testing as part of this method. Differences in GM composition across predictor variables were visualised via a Principal Components Analysis (PCA). A second analysis was also performed using ASV abundances that were instead transformed using the Phylogenetic Isometric Log Ratio transformation (PhILR) in the R package *philr* 1.14.0 [80]. This transformation controls for the compositionality of the data but also preserves information about the relatedness of ASVs, thus providing a phylogenetically aware measure of beta diversity [80].

##### Body condition

Samples were filtered as above (see alpha diversity analysis), leaving a total of 425 samples from 296 individuals. As GM composition differed across age classes and adult birds were found to be significantly heavier than juvenile individuals (fledgling, old fledgling and sub-adult age classes, Additional file 1: Table S2), subsequent analyses were carried out separately for juveniles (205 samples from 175 individuals) and adults (220 samples from 165 individuals); this was to ensure that any confounding influence of age class and body condition on GM composition could be separated. For juvenile age classes, a PERMANOVA analysis was conducted using a Euclidean distance matrix of CLR-transformed ASV abundances, with 9,999 permutations. Body condition was included as a predictor variable, by extracting the residuals of a regression of body mass on tarsus length, controlling for the time of day, separately for males and females. Individual sex, territory quality and the corresponding sampling field period (Major 2017, Minor 2018, Major 2018, Minor 2019, Major 2019, Minor 2020) were included as additional predictors. Age class (fledgling, old fledgling or sub-adult) was also included in the juvenile model. Bird ID was included as a blocking factor to control for repeated sampling. A second analysis was also performed using ASV abundances that were instead transformed using the PhILR transformation [80].

##### Survival

To identify compositional differences in the GM between individuals that survived versus those that died, sequencing and catch duplicates were removed, as were samples taken from nestlings, floaters and those collected in 2020. The remaining samples were also filtered to retain the latest sample per individual (as described above). Post-filtering, 264 samples containing 2,900 ASVs were retained (226 individuals that survived, 38 individuals that died). As for the analysis of beta diversity and body condition, analyses were carried out separately for juveniles (116 samples/individuals; 17 died, 99 survived) and adults (148 samples/individuals; 21 died, 127 survived), since survival probability in the Seychelles warbler is significantly lower in the first year of life (encompassing fledglings to sub-adults) [44]; as such, separating juveniles and adults ensures that changes in GM structure that are associated with age and survival are not confounded. For each age group (juvenile or adult) a PERMANOVA analysis was performed, using a Euclidean distance matrix of CLR-transformed ASV abundances, with 9,999 permutations. Survival to the next breeding season (yes, no), sex, territory quality and the corresponding sampling field period (Major 2017, Minor 2018, Major 2018, Minor 2019, Major 2019) were included as predictors. Age class (fledgling, old fledgling or sub-adult) was also included in the juvenile model. Differences in GM composition across samples were visualised via PCA. As above, a second analysis was also performed using a Euclidean distance matrix calculated from PhILR-transformed ASV abundances to assess whether communities were phylogenetically distinct across groups.

To establish whether specific ASVs were differentially abundant across groups of individuals, an Analysis of Compositions of Microbiomes with Bias Correction (ANCOM-BC) was carried out, using the *ANCOMBC* 1.1.5 package in R [81]. Differential abundance was tested between groups of adult individuals that survived, versus those that died, whilst controlling for sex and sampling season. As part of ANCOM-BC, the Benjamini and Hochberg method was used to correct *P* values for multiple testing [66]. A cut-off of *P*_*adj*_ < 0.05 was used to assess significance.

## Results

### Gut microbiome variation

Following the removal of control samples and those that had fewer than 10,000 reads, the number of high-quality reads per sample ranged from 10,979 to 744,600, across 586 faecal samples. Reads were clustered into 55,664 ASVs, with a mean of 383 ± 277 (SD) ASVs per sample. Both alpha and beta diversity metrics showed high levels of similarity across extraction and sequencing repeats, with pairwise Euclidean distances between samples extracted and/or sequenced twice being significantly lower than those measured between pairs of different samples (*P*_*adj*_ < 0.01 in bootstrapped Dunn’s multiple comparison tests, Additional file 1: Fig. S2). Following the removal of extraction, sequencing, and catch duplicates, 470 samples remained which contained a mean of 368 ± 253 (SD) ASVs per sample after rarefying to 10,000 reads.

**Fig. 1:**
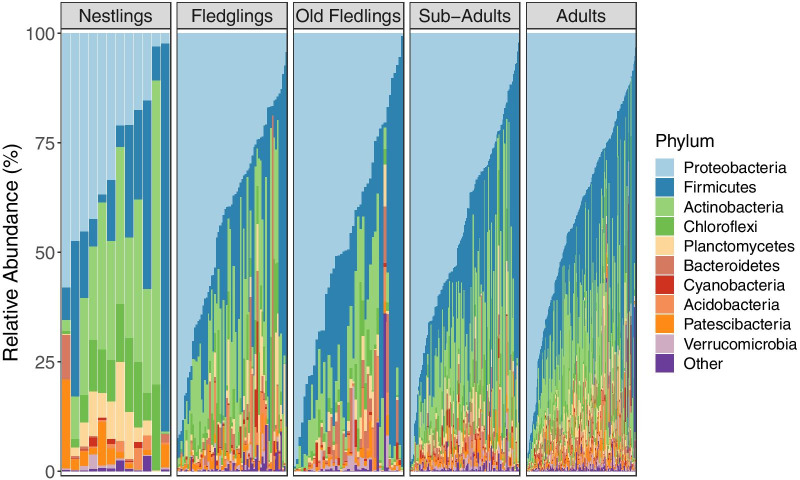
The relative abundance (%) of bacterial phyla in Seychelles warbler gut microbiome samples. Each vertical bar represents a separate faecal sample. Samples are categorised by age class (nestling, fledgling, sub-adult, or adult) and are ordered according to the abundance of *Proteobacteria*. N = 470 samples from 370 individuals in total: nestlings = 12, fledglings = 65, old fledglings = 45, sub-adults = 107 and adults = 241 samples, respectively. Phyla with a median relative abundance of less than 1% are collapsed into the category “Other”

Consistent with a previous study on the Seychelles warbler [45], faecal samples were dominated by the phyla *Proteobacteria* (mean relative abundance = 43% ± 24% SD), *Firmicutes* (26% ± 24%), and *Actinobacteria* (15% ± 13%). For full details of the core microbiome see [45]. However, despite the dominance of these taxa, there was also substantial inter-individual variation in GM composition (Fig. 1).

A PERMANOVA analysis revealed that GM composition differed significantly between individuals in different host age classes (Table 1). However, this was only when CLR- (and not PhILR-) transformed ASV abundances were used (CLR PERMANOVA: F_3,446_ = 1.320, *P* = 0.004; PhILR PERMANOVA: F_3,446_ = 1.721, *P* = 0.067, Table 1), indicating that although GM composition differed, the bacterial communities weren’t phylogenetically distinct across the different age classes. A post-hoc pairwise PERMANOVA analysis using the CLR-transformed abundances indicated that fledglings had a significantly different GM composition compared to old fledglings and adult birds (*P*_adj_ < 0.05, Additional file 1: Table S1). A betadisper analysis indicated that there were also differences in GM variability across age classes (CLR betadisper: *F*_3,446_ = 6.062, *P* < 0.001). Indeed, a PCA analysis demonstrated that although clusters overlapped for all age classes, the GM of sub-adults had slightly greater levels of variation overall (Additional file 1: Fig. S3).

**Table 1:** PERMANOVA analysis of gut microbiome distances in the Seychelles warbler

Predictor	*df*	*R*^2^		*F*		*P*	
		CLR	PhILR	CLR	PhILR	CLR	PhILR
Age class	3	0.008	0.011	1.320	1.721	**0.004**	0.067
Sex	1	0.003	0.006	1.635	2.760	**0.013**	**0.038**
Territory quality	1	0.003	0.004	1.271	1.720	0.244	0.350
Sampling period	5	0.043	0.051	4.077	4.874	**< 0.001**	**< 0.001**

Euclidean distances were calculated based on either CLR or PhILR transformed Amplicon Sequencing Variant (ASV) abundances. Significant predictors (*P* < 0.05) are shown in bold. The analysis included 450 samples from 309 individuals. Bird ID was included as a blocking factor to control for the repeated sampling of individuals

In addition to age class, host sex had a significant effect on GM structure (CLR PERMANOVA: *F*_1,448_ = 1.635, *P* = 0.013; PhILR PERMANOVA: F_1,448_ = 2.760, *P* = 0.038, Table 1, Additional file 1: Fig. S4), although it explained less than 0.6% of the variation in GM beta diversity (R^2^ = 0.006, Table 1). A betadisper analysis further indicated that the GM composition of females was slightly more variable compared to males (CLR betadisper: *F*_1,448_ = 5.077, *P* = 0.025; PhILR betadisper: *F*_1,448_ = 5.643, *P* = 0.018).

**Fig. 2:**
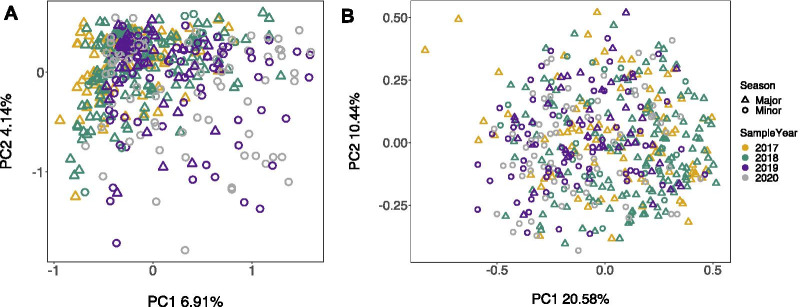
Variation in gut microbiome composition across sampling periods in the Seychelles warbler. Principal Components Analysis (PCA) of Euclidean distances calculated using **A** CLR-transformed ASV abundances or **B** PhILR-transformed abundances. Each point represents a unique gut microbiome sample (N = 450). Samples were taken from 309 individuals. Principal components one and two explained 6.91% and 4.14% of the variation in gut microbiome structure in the CLR analysis, and 20.58% and 10.44% in the PhILR analysis, respectively

The beta diversity of GM samples also differed significantly across sampling periods (Table 1, Fig. 2A). Post-hoc pairwise PERMANOVA tests confirmed that the GM was compositionally distinct between all sampling periods when analysing CLR-transformed ASV abundances (*P* < 0.05; Additional file 1: Table S1). Similarly, the phylogenetic structure of GM samples differed significantly across sampling periods (Table 1, Fig. 2B). However, pairwise tests showed that not all sampling periods were phylogenetically distinct (*P* > 0.05; Additional file 1: Table S1) for example, the minor period of 2019 was phylogenetically similar to the consecutive minor periods of 2018 and 2020, respectively (Additional file 1: Table S1). Differences in GM variability were also identified across the different sampling periods when analysing CLR-transformed abundances (CLR betadisper: *F*_5,444_ = 9.394, *P* < 0.001, Additional file 1: Fig. S5); there was a slight increase in variability in the minor sampling periods of 2019 and 2020 and lower variability in the major sampling period of 2019 (Additional file 1: Fig. S5). None of the sampling periods were phylogenetically more variable than others (PhILR betadisper: *F*_5,444_ = 1.746, *P* = 0.123). Sampling period explained the largest amount of variation in GM composition across individuals (up to 5% of the total variation; R^2^ = 0.043 and 0.051 in CLR and PhILR PERMANOVAs respectively, Table 1), with all other variables explaining a smaller proportion of the overall variance (Table 1). Territory quality, which varies across the island, had no influence on GM structure (CLR PERMANOVA: *F*_1,448_ = 1.271, *P* = 0.244; PhILR PERMANOVA: F_1,448_ = 1.720, *P* = 0.350, Table 1).

### Gut microbiome diversity and body condition

Samples were next filtered so that only those taken from individuals with a corresponding measure of body condition were retained. There was no significant association between the alpha diversity of GM samples (Shannon diversity) and individual body condition, measured as size-corrected body mass (*P*_*adj*_ = 0.963, Additional file 1: Table S2). Results were very similar when Chao1 richness or Faith’s PD were used as the alpha diversity metric in models (Additional file 1: Table S2). As shown previously [68], body condition was significantly greater in males and adult individuals (Additional file 1: Table S2). Body condition also increased significantly over the course of the day and varied across the different sampling field periods (Additional file 1: Table S2).

PERMANOVA analyses were carried out to establish whether differences in GM composition were associated with variation in body condition across individuals. To separate the influence of age and body condition on GM structure, analyses were conducted separately for juvenile (fledglings, old fledglings and sub-adults) and adult individuals; this is because, as noted above, body condition (size-corrected body mass) was found to be significantly greater in adults compared to juvenile age classes (Additional file 1: Table S2) and GM composition also varies according to host age class (Table 1). There was no significant relationship between GM composition and body condition in juveniles (CLR PERMANOVA: *F*_1,203_ = 1.600, *P* = 0.262; PhILR PERMANOVA: F_1,203_ = 1.998, *P* = 0.229; Additional file 1: Table S3). This was also the case for adult individuals (CLR PERMANOVA: *F*_1,218_ = 0.974, *P* = 0.788; PhILR PERMANOVA: F_1,218_ = 0.666, *P* = 0.538; Additional File 1: Table S3). Together, these results suggest that there is no association between GM community structure and individual body condition in the Seychelles warbler.

### Gut microbiome alpha diversity and survival

There was no significant association between GM alpha diversity (measured as Shannon diversity) and the probability that an individual survived to the next breeding season (*P*_*adj*_ = 0.888, Table 2). This result was robust, regardless of whether Shannon diversity, Chao1 richness or Faith’s PD were used as alpha diversity metrics (Additional file 1: Table S4). Old fledglings (3–6 months old) had a slightly lower probability of survival compared to adult individuals (*P*_*adj*_ = 0.042, Table 2; Additional file 1: Table S4 and Fig. S6), which is consistent with previous findings in the Seychelles warbler [44].

**Table 2:** A Generalised Linear Model investigating the association between gut microbiome alpha diversity (Shannon diversity) and survival in the Seychelles warbler

Predictor	Estimate	SE	*z*	*P*_*adj*_
**Intercept**	**1.515**	**0.610**	**2.483**	**0.013**
Shannon	− 0.117	0.378	− 0.308	0.888
Age class				
Fledgling	− 0.275	0.564	− 0.487	0.626
** Old fledgling**	− **1.073**	**0.503**	− **2.134**	**0.042**
Sub-adult	0.954	0.583	1.637	0.123
Sex (male)	− 0.484	0.372	− 1.300	0.230
Territory quality	1.246	0.699	1.782	0.075
Sample year				
2018	0.599	0.724	0.827	0.408
2019	0.326	0.744	0.439	0.661

Significant (*P*_*adj*_ < 0.05) predictors are shown in bold; *P* values were corrected for multiple hypothesis testing using the Benjamini and Hochberg method- this was to control for the use of different alpha diversity metrics (results for other metrics are shown in Additional file 1: Table S4). Reference categories for categorical variables were as follows: adult (age class), female (sex) and 2017 (sample year). *N* = 264 samples/individuals were included in the analysis (226 individuals survived, 38 individuals died by the next breeding season)

### Gut microbiome beta diversity and survival

**Fig. 3:**
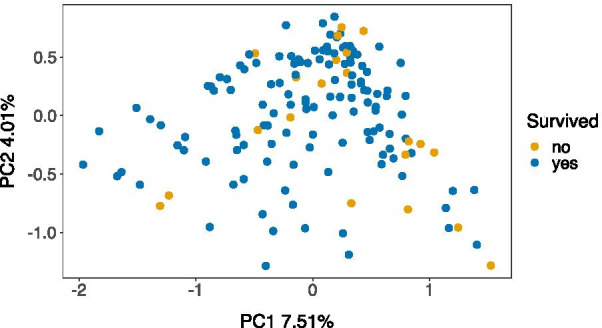
Principal components analysis (PCA) of Euclidean distances between the gut microbiomes of adult Seychelles warblers that survived (blue) versus those that had died (yellow) by the next breeding season. Each point represents a sample taken from a different individual. Euclidean distances are based on CLR-transformed abundances of ASVs. Principal components one and two explained 7.51% and 4.01% of the variation in GM community structure, respectively. *N* = 148 samples/individuals were included in the analysis (127 individuals survived, 21 individuals died)

To identify whether there were compositional differences in the GM between individuals that survived versus those that died by the next breeding season PERMANOVA analyses were carried out; these were conducted separately for juvenile and adult age classes, to avoid confounding the influence of age with differential survival (since mortality is highest in juvenile warblers [44]). Across juveniles, there was no significant association between GM composition and survival to the next breeding season (CLR PERMANOVA F_1,114_ = 1.033, *P* = 0.334; PhILR PERMANOVA F_1,114_ = 1.360, *P* = 0.138; Table 3A). However, for adults, GM composition differed significantly between individuals that survived versus those that had died by the next breeding season (CLR PERMANOVA F_1,146_ = 1.313, *P* = 0.032, Table 3B, Fig. 3). In this instance, survival explained 1% of the variation in GM community structure across individuals (R^2^ = 0.009, Table 3B). Importantly, a betadisper test showed that the PERMANOVA result was caused by differences in the mean location of samples rather than differences in GM variability between the two adult groups (CLR betadisper: *F*_1,146_ = 0.253, *P* = 0.614). Differences in GM phylogenetic structure between adult birds that survived versus those that died were marginally insignificant (PhILR PERMANOVA F_1,146_ = 1.650, *P* = 0.058, Table 3B).

**Table 3:** PERMANOVA analysis of gut microbiome distances and survival in (A) juvenile and (B) adult Seychelles warblers

Predictor	*df*	*R*^*2*^		*F*		*P*	
		CLR	PhILR	CLR	PhILR	CLR	PhILR
(A) Juveniles							
Age class	2	0.018	0.018	1.077	1.090	0.185	0.295
Sex	1	0.009	0.011	1.130	1.378	0.138	0.140
Territory quality	1	0.008	0.008	1.001	1.024	0.439	0.378
**Sampling period**	4	0.060	0.079	1.802	2.416	**< 0.001**	**< 0.001**
Survival	1	0.009	0.011	1.033	1.360	0.334	0.138
(B) Adults							
Sex	1	0.008	0.009	1.184	1.451	0.084	0.106
Territory quality	1	0.007	0.005	1.067	0.824	0.282	0.633
**Sampling period**	4	0.062	0.080	2.366	3.139	**< 0.001**	**< 0.001**
**Survival**	1	0.009	0.011	1.313	1.650	**0.032**	0.058

Euclidean distances were calculated based on either CLR or PhILR transformed Amplicon Sequencing Variant (ASV) abundances. Significant predictors (*P* < 0.05) are shown in bold. Analyses included 116 juvenile individuals (17 died, 99 survived) and 148 adults (21 died, 127 survived), respectively

### Bacterial taxa associated with survival in adults

**Fig. 4:**
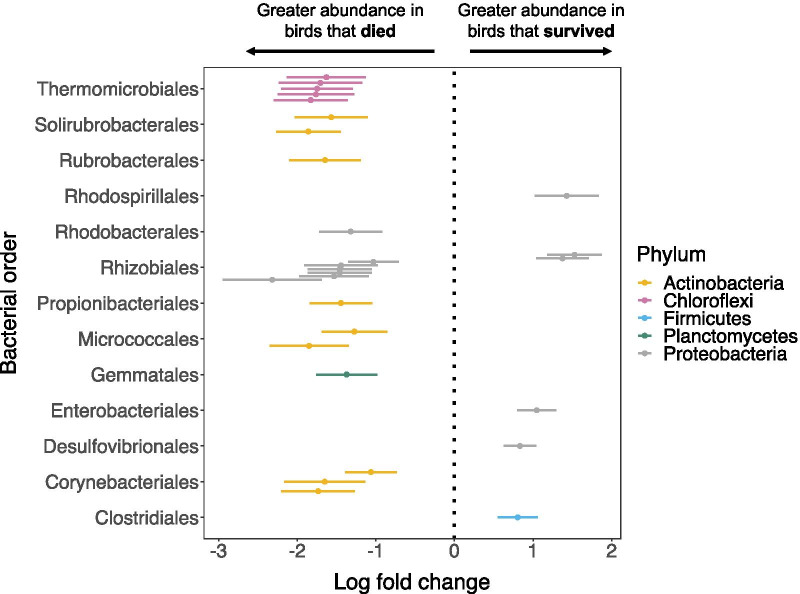
Differentially abundant Amplicon Sequencing Variants (ASVs) in the gut microbiome of adult Seychelles warblers that survived versus those that died by the next breeding season. *N* = 148 adult individuals were included in the analysis (127 individuals survived, 21 individuals died). Points represent the log fold change (effect size) of individual bacterial ASVs—only those with significant effect sizes (*P*_adj_ < 0.05) are shown. A positive log fold change indicates that an ASV is more abundant in individuals that survived (right), and a negative log fold change indicates a higher abundance in individuals that died by the next season (left). Bars represent 95% confidence intervals derived from the ANCOM-BC model. ASVs are classified by bacterial order on the y-axis and are coloured by phylum. Results of differential abundance tests and ASV taxonomies are presented in full in Additional file 1: Table S5

There were 28 bacterial ASVs significantly (*P*_*adj*_ < 0.05, ANCOM-BC) differentially abundant between the GM of adult individuals that survived versus those that died by the next breeding season (Fig. 4, Additional file 1: Table S5). Of these, six ASVs were significantly more abundant in individuals that survived (Fig. 4, Additional file 1: Table S5). These were members of two phyla, namely *Proteobacteria* (five ASVs) and *Firmicutes* (one ASV). The enriched *Proteobacteria* included one ASV from the order *Desulfovibrionales* (family *Desulfovibrionadaceae*, genus *Desulfovibrio*), two ASVs in the order *Rhizobiales* (family *Rhizobiaceae*, genus *Bartonella),* one in the order *Enterobacteriales* (family *Enterobacteriaceae*, genus *Pragia*) and one in the order *Rhodospirillales* (family *Rhodospirillaceae*, genus *Pararhodospirillum*) (Additional file 1: Table S5). The enriched ASV in the phylum *Firmicutes* was in the order *Clostridiales* (*Clostridiales* family XIII, genus *Anaerovorax*) (Additional file 1: Table S5).

The 22 ASVs that were identified as being more abundant in individuals that had died by the following breeding season belonged to four different phyla, namely *Planctomycetes* (one ASV, uncultured member of the family Gemmataceae), *Chloroflexi* (five ASVs), *Actinobacteria* (nine ASVs) and *Proteobacteria* (seven ASVs) (Fig. 4, Additional file 1: Table S5). All ASVs in the phylum *Chloroflexi* were uncultured members of the bacterial order *Thermomicrobiales* (Additional file 1: Table S5). The enriched Actinobacterial ASVs were classified into the orders *Propionibacteriales* (one ASV, genus *Microlunatus*), *Solirubrobacterales* (two ASVs, uncultured genus), *Rubrobacteriales* (one ASV, genus *Rubrobacter*), Microccales (two ASVs in the genera *Kocuria* and *Microbacterium*, respectively) and *Corynebacteriales* (three ASVs, all in the genus *Mycobacterium*) (Additional file 1: Table S5). The enriched ASVs in the phylum *Proteobacteria* were classified in the orders *Rhizobiales* (two uncultured genera, one *Methylobacterium* and three ASVs in the genus *Aureimonas*) and *Rhodospirillales* (genus *Rubellimicrobium*) (Additional file 1: Table S5).

## Discussion

In this study, we use data from a closed, island population of Seychelles warblers, to investigate the association between GM variation, host condition, and survival. Results show that there is substantial variation in GM diversity across individuals within the population on Cousin Island. The composition of the GM was associated with seasonal variation and, to a lesser extent, with an individual’s sex and age. While there was no association between GM alpha diversity and body condition or survival, we did identify significant differences in GM composition between adult individuals that survived versus those that died by the next breeding season, with several bacterial taxa being differentially abundant between the two groups.

### Gut microbiome diversity and body condition

Previous studies investigating the relationship between GM alpha diversity and individual condition have shown mixed results, with both positive [24, 32, 82] and negative [33, 34, 83] relationships, as well the absence of an association [84, 85] being identified across various wild and captive vertebrate host species. However, these studies often focus on early life stages, despite the fact that body mass can be an important predictor of fitness in adult individuals [68, 86, 87]. Additionally, several of these studies used antibiotics to artificially alter GM diversity and composition [33, 83] and so it is unclear how well these relationships hold in natural, unmanipulated populations. We found no relationship between body condition and GM alpha diversity in the Seychelles warbler, sampled across four different age classes post-fledging. Furthermore, the relationship between GM beta diversity and body condition was not significant in adult or juvenile individuals. Additionally, there was no relationship between GM beta diversity and body condition when taking the phylogenetic relatedness of ASVs into account. This suggests that individuals with different body condition did not carry consistently different, or phylogenetically distinct, bacterial communities.

There are several possible explanations for the lack of an association between GM characteristics and body condition (measured as size-corrected body mass) in the Seychelles warbler. Birds (and other flying organisms) are under strong selection for lower body mass to improve flight efficiency [88]; it has been suggested that this pressure may extend to the need to reduce microbial biomass in the intestinal tract [89]. Indeed, many bird species have reduced gut lengths and shorter food retention times compared to non-flying vertebrates [90]. An increased rate of intestinal paracellular absorption compensates for this by enabling greater quantities of simple nutrients to be absorbed by the bird’s own cells [90]. Together, these adaptations may have reduced reliance on microbial metabolism and, consequently, the potential for bacteria in the gut to strongly influence physical traits that impact flight in birds—such as body mass [89]. This may be particularly pertinent in the Seychelles warbler as they glean insects from the undersides of leaves whilst in flight, and therefore require high flight efficiency. A study on great tits also failed to find a direct association between nestling body mass and GM alpha diversity, but identified a time-lagged relationship whereby nestling weight at day eight was negatively associated with GM alpha diversity at day fifteen [34]. It is possible that such a relationship exists in the Seychelles warbler, however a lack of faecal samples from nestlings and difficulties in catching the same individual within a short timeframe meant that it was not possible to test for this. Since individuals in ill health may also be less active, and thus more difficult to catch in mist nets, we also acknowledge that individuals in very poor condition, which may experience more extreme GM deviations, may not be represented in the dataset.

The relationship between microbial diversity metrics and emergent properties of microbial communities, such as functional capacity, productivity and stability, can be highly complex [46, 91]. For example, microbial communities with very different alpha diversities can have similar functional capacities [91]. Similarly, greater numbers of transitionary microbes could add to GM diversity but contribute very little in terms of long-term benefits to the host, such as increasing energy availability or enhancing host immunity [92]. It has been suggested that the reduced complexity and specificity of the bird digestive system, compared to mammalian species, may increase the abundance and variety of transitionary gut microbes [89, 92, 93]. In support of this, a study on New Guinean birds demonstrated that the GM of smaller passerine species was less stable and more heterogenous than that of larger species, presumably because shorter guts and faster retention times can result in stronger ecological drift and a higher turn-over of bacterial species acquired from environmental sources [94]. As Seychelles warblers are insectivorous, bacterial species could be readily acquired from their insect prey [95] as well as from the surrounding environment. As such, differences in GM diversity across individuals could potentially reflect variable uptake from these sources. The significant influence of sampling period on GM composition in the Seychelles warbler further indicates that this could be the case. Thus, the expectation that high GM alpha diversity is beneficial is over-simplified and may not always extend from laboratory studies, in which environmental conditions are highly controlled and homogenous [46, 96].

Functional redundancy can also complicate relationships involving beta diversity, since GM communities with different compositions may be capable of performing the same set of functions and, as such, could influence the host phenotype in similar ways [97]. Such complexities could be hidden in analyses involving beta diversity metrics. An assessment of bacterial function, via metagenomic sequencing, may give further insight into whether GM functional diversity, rather than differences in the number or identity of species, is a more important metric for determining host condition. Other measures of host condition could also be incorporated into future analyses. For example, deviations in white blood cell populations have previously been used to assess host health status in a study on northern elephant seals (*Mirounga angustirostris*); these were in turn linked to differences in GM diversity [24]. Haematocrit (the proportion of blood comprising of erythrocytes) has previously been shown to be linked to the condition and survival of Seychelles warblers [98] and, thus, could be a useful alternative metric in future studies.

### Gut microbiome variation and survival

Consistent with the body condition analysis, we found no relationship between GM alpha diversity and survival in the Seychelles warbler. Similarly, there was no association between GM composition and survival in juvenile birds. However, small differences in GM composition were identified between adult individuals that survived to the next breeding season and those that did not. Juvenile and adult warblers are exposed to differing stressors which may alter the relative impact of the GM on survival. For example, juvenile individuals often become infected with the haemosporidian blood parasite, *Haemoproteus nucleocondensus,* which may lead to greater mortality in this age group [99, 100]*.* Infection prevalence peaks in the first year of life (84% in juveniles), before declining in adulthood as surviving individuals either suppress or clear the infection [99]. Furthermore, juveniles gradually become less dependent on their parents for food and may disperse from their natal territories; at this point, they experience intense competition for resources which may also impact upon their survival [44, 101]. Thus, mortality in juveniles may be influenced by a variety of factors that may act independently of the GM. Adults experience these pressures to a lesser extent; changes in the GM may therefore have a greater relative impact on the adult life stage.

Differential abundance analysis identified 28 ASVs that were significantly differentially abundant between adults that survived versus those that died. Few studies have investigated the role that particular bacterial species play in the GM of wild vertebrates and so extrapolating the function of differentially abundant taxa is often difficult and highly speculative [102–104]. However, there were several differentially abundant taxa that are known to be common members of the vertebrate gut and may potentially play a role in host health and functioning. For example, one member of the order *Clostridiales* was more abundant in the GM of adult individuals that survived. Members of the order *Clostridiales* are abundant in the GM of many vertebrate taxa, including other insectivorous passerine species [105, 106] and have previously been linked to an increase in immunological resistance to nest parasites in eastern bluebirds (*Sialia sialis*) [107]. A study on captive, juvenile ostriches (*Struthio camelus*), also showed that the abundance of ASVs in this order was reduced in the hindgut of diseased individuals that subsequently died, suggesting that they may be linked to host health and survival [11]. Species in the order *Clostridiales* play a role in carbohydrate and protein fermentation (for example during the digestion of insect prey) as well as the degradation of toxic by-products from this process [105, 108]. The short-chain fatty acids produced from fermentation can be directly absorbed across the intestinal wall and used as an energy source by the host [102]. Butyrate is one such end-product and plays an important role in maintaining colonic health in humans and other laboratory organisms [109]. A member of the genus *Desulfovibrio* was also enriched in adult Seychelles warblers that survived. *Desulfovibrio* are sulphate-reducing bacteria that are common in the human gut microbiome [110]; they consume hydrogen, which is a by-product of protein fermentation and, in doing so, increase the energy yields achieved from this process [110, 111].

In contrast, three ASVs in the genus *Mycobacterium* were more abundant in the GM of adult individuals that had died by the next breeding season. The genus *Mycobacterium* includes several pathogenic species that are known to be the causative agents of diseases, such as tuberculosis, in vertebrate taxa. In birds, avian tuberculosis is primarily an intestinal disease, caused by strains in the *Mycobacterium avium* species complex [112, 113]. It has a prolonged incubation period, with the infection developing over several weeks and months, leading to symptoms such as intestinal inflammation, diarrhea and weakness [112]. It is acquired from the environment and can be transmitted via faecal droppings [112, 113]. Despite the implication that this genus is involved in avian disease and could contribute to mortality in the Seychelles warbler, further study is needed to taxonomically identify these ASVs beyond genus level and to confirm their pathogenicity.

In addition to species of *Mycobacterium*, several species in the order *Thermomicrobiales* (phylum *Chloroflexi*) were enriched in the GM of individuals that died by the next breeding season. Although many species in the phylum *Chloroflexi* are poorly characterised, they are distributed across a wide range of environments including freshwater, brackish and marine habitats [114]. Members of this phylum have also been identified at low abundances in the mammalian GM and, in some cases, have been shown to proliferate in diseased humans [114, 115]. Since the ASVs in the order *Thermomicrobiales* were also present at lower abundances in warbler individuals that subsequently survived to the next breeding season, these ASVs may have proliferated in adult individuals which died shortly after sampling. However, further functional characterisation will be needed to confirm the role that these bacteria play in the GM of avian host species.

One member of the order *Propionibacteriales* was also more abundant in adult individuals that died. While members of this order occur in a diverse range of habitats, and are commensals in the GM of various vertebrate species, they are also facultative parasites, at least in humans [116]. Similarly, three ASVs in the genus *Aureimonas* (order Rhizobiales) were enriched in adult birds that died; members of this genus have primarily been isolated from environmental sources, but there are indications that certain species can be pathogenic to humans [117, 118]. It is important to note here, that changes in ASV abundances within the GM of the Seychelles warbler (e.g., increased abundances of pathogenic species, or reduced abundances of beneficial species) could be causally linked to the death of individuals. However, equally, observed differences could be the outcome of GM perturbations resulting from a decline in health, or changes in host physiology, close to death. Thus, an enrichment of certain ASVs could be a by-product of the processes linked to death, rather than a cause of death, although these are not necessarily mutually exclusive. Functional evaluation of bacterial species and experimental manipulation of the microbiome would be needed to confirm which of these was the case.

Although there were significant differences between the GM of adult individuals that survived and those that died, survival only explained a small percentage (1%) of the overall variation in GM composition across adult individuals. For 14 of the 21 adult individuals that died by the next breeding season, the date of GM sampling was the last time they were observed in the population. However, the remaining seven individuals that died were observed (but not sampled) again in the same breeding season as GM sampling took place; in some cases, this was up to eleven weeks after their last GM sample was taken suggesting they had remained alive for a substantial period following sampling. Additionally, there was a median period of five months between the point when these adult GM samples were taken, and when the population was next censused to assess survival. Thus, it is possible that some of the individuals were sampled up to five months before their point of death, when only small differences, or imbalances, in the GM may have been detectable. A study on survival in juvenile, captive ostriches showed that, although there was a correlation between the diversity of the GM during the first weeks of life and the probability of survival beyond six weeks of age, the relationship was strongest in the weeks closest to death [11]. Thus, greater differences might be expected in the months or weeks immediately before death, either as a result of pathogen proliferation or further GM disruption caused by a decline in health. However, we should also acknowledge that the primary cause of death in the Seychelles warbler is largely unknown and so such a relationship may not be the case, or could be further diluted, if death was the result of stochastic events for most individuals, such as entanglement with *Pisonia* seeds or injury. A further possible explanation for the low levels of explained variance (for survival and other terms investigated in this study), is the greater abundance and variety of transitionary microbes that are expected to pass through the avian digestive system as a result of shorter intestinal lengths [94]; these transient colonisers may add a greater level of noise to analyses investigating the factors that influence GM composition in birds.

### Host and environmental factors influencing gut microbiome beta diversity

In addition to survival status, other factors were also found to significantly influence GM beta diversity across Seychelles warblers in our study, including the age of the individual. Fledglings had a significantly different GM composition compared to other age classes, despite the fact that they are still reliant on food from their parents and remain in their natal territory. Development has been shown to strongly influence GM composition in humans and other primates [29, 119] and, although few studies have investigated changes in the GM across the life course of birds, several studies have identified differences between the GM of nestlings versus adult individuals [82, 120]. Sub-adult Seychelles warblers also had a more variable GM community compared to other age classes. Birds in this age class are no longer dependent upon their parents for food, but instead are learning to feed themselves. They may also leave their natal territory at this point [101]. Thus, differences in their GM could reflect a reduction in food quality as they become independent, or greater exposure to environmental variation [45].

We also identified significant differences in GM composition and phylogenetic structure between the sexes in the Seychelles warbler. Reproductive physiology differs between male and female animals and this can manifest in different GM profiles [102]. For example, the reproductive hormone testosterone is thought to be an immunosuppressant [121]. As such, the concentration of circulating testosterone has been shown to positively correlate with bacterial diversity and the relative abundance of *Chlamydia* species in the cloacal microbiome of male rufous-collared sparrows (*Zonotrichia capensis*) [122]. Although there were significant dissimilarities in GM structure between the sexes in the Seychelles warbler, sex only explained a small percentage (< 0.6%) of the overall variation in GM composition across individuals. The extent to which sex drives differences in the GM varies substantially across wild vertebrate populations [e.g. 21, 26, 123], but greater differences are often seen in highly dimorphic species [24]. Male and female Seychelles warblers share the same diet and exhibit relatively low levels of morphological and behavioural dimorphism, potentially explaining the relatively weak contribution of sex to GM variation in this system.

Sampling period explained the largest proportion of variation (4.3–5.1%) in GM composition across the individuals sampled in this study. Climatic variables, such as rainfall, can vary substantially between years and seasons on Cousin Island, which could impact upon the microbial species present in the external environment. Climatic variables could also influence the island-wide abundance, type, or quality of insect prey between seasons, although, at a local-scale, variation in territory qualities across the island had no influence on GM structure. Season has been identified as an important factor driving differences in the GM of many other wild animal species [25–27] and may be particularly important in avian species that have fast intestinal retention times and a higher turn-over of transitionary bacterial species that are acquired from their environment [94]. Dietary differences have been identified as a key driver of seasonal variation in mammalian species [27] and can lead to significant shifts in the GM composition of birds [105, 107, 124].

## Conclusions

Few studies have investigated the association between the GM and fitness components in wild animal populations, yet such studies are necessary if we are to assess the evolutionary role of the GM. The Seychelles warbler represents an excellent system in which to study the relationship between GM variation and fitness, since survival and life history parameters can be accurately measured for individuals across all age classes. In our study, we show that GM variation was not associated with body condition in the Seychelles warbler. However, while GM alpha diversity was not associated with survival, we identified significant differences in the composition of the GM between adult individuals that survived, versus those that died, although this was not the case in juvenile birds. Adult birds that died carried reduced abundances of potentially beneficial bacterial taxa but had greater abundances of bacterial taxa that have previously been identified as opportunistic pathogens in birds and other systems. To our knowledge, this is the first time that GM differences associated with survival have been fully characterised for a wild vertebrate species, across multiple age groups and seasons. Future assessments of the functional diversity of the GM will be crucial for understanding the potential contribution of differentially abundant bacterial taxa to avian health. Studying the link between GM characteristics and other fitness components, such as reproductive success, will also provide further insight into the evolutionary significance of GM variation.

## Supplementary Information

**Additional file 1.** Supplemental tables and figures referenced in the text.** Fig. S1**. Sample completeness curves.** Fig. S2**. The similarity of A) alpha, and B) beta diversity measures across Seychelles warbler faecal samples.** Table S1**. The results of post-hoc pairwise PERMANOVA analyses investigating differences in gut microbiome composition across A) age classes and B) sampling periods.** Fig. S3**. Differences in gut microbiome (GM) composition across age classes in the Seychelles warbler.** Fig. S4**. A Principal Components Analysis (PCA) of Euclidean distances between the gut microbiome (GM) of male and female individuals.** Fig. S5**. Results of a betadisper analysis showing differences in gut microbiome variability (distance to centroid) across sampling periods.** Table S2**. Linear Mixed Model analyses investigating the association between gut microbiome alpha diversity and body condition in the Seychelles warbler.** Table S3**. PERMANOVA analysis of gut microbiome distances and body condition in A) juvenile and B) adult Seychelles warblers.** Table S4**. Generalised Linear Model investigating the association between gut microbiome alpha diversity and survival in the Seychelles warbler.** Fig. S6**. Survivorship across different bird age classes.** Table S5**. Amplicon sequencing variants (ASVs) that were significantly, differentially abundant (*P*_*adj*_ < 0.05) in the gut microbiomes of adult Seychelles warbler individuals that survived, versus those that died, by the next breeding season.

## Data Availability

All 16S rRNA gene amplicon sequences have been submitted to the European Nucleotide Archive (ENA) database under the study accession numbers PRJEB45408 (samples taken in 2017 and 2018) and PRJEB47095 (samples taken in 2019 and 2020). The scripts and metadata to reproduce all analyses and figures can be accessed via the GitHub repository, https://github.com/Seychelle-Warbler-Project.
