# Supplementary material for: Gut microbiome composition, not alpha diversity, is associated with survival in a natural vertebrate population
Source: Anim Microbiome. 2021 Dec 20;3:84. doi: 10.1186/s42523-021-00149-6 (PMC8685825; doi:10.1186/s42523-021-00149-6)
Supplement: Supplementary file 1 — Additional file 1. Supplemental tables and figures referenced in the text. Fig. S1. Sample completeness curves. Fig. S2. The similarity of A) alpha, and B) beta diversity measures across Seychelles warbler faecal samples. Table S1. The results of post-hoc pairwise PERMANOVA analyses investigating differences in gut microbiome composition across A) age classes and B) sampling periods. Fig. S3. Differences in gut microbiome (GM) composition across age classes in the Seychelles warbler. Fig. S4. A Principal Components Analysis (PCA) of Euclidean distances between the gut microbiome (GM) of male and female individuals. Fig. S5. Results of a betadisper analysis showing differences in gut microbiome variability (distance to centroid) across sampling periods. Table S2. Linear Mixed Model analyses investigating the association between gut microbiome alpha diversity and body condition in the Seychelles warbler. Table S3. PERMANOVA analysis of gut microbiome distances and body condition in A) juvenile and B) adult Seychelles warblers. Table S4. Generalised Linear Model investigating the association between gut microbiome alpha diversity and survival in the Seychelles warbler. Fig. S6. Survivorship across different bird age classes. Table S5. Amplicon sequencing variants (ASVs) that were significantly, differentially abundant (Padj < 0.05) in the gut microbiomes of adult Seychelles warbler individuals that survived, versus those that died, by the next breeding season. [file 42523_2021_149_MOESM1_ESM.pdf]

Additional File 1:

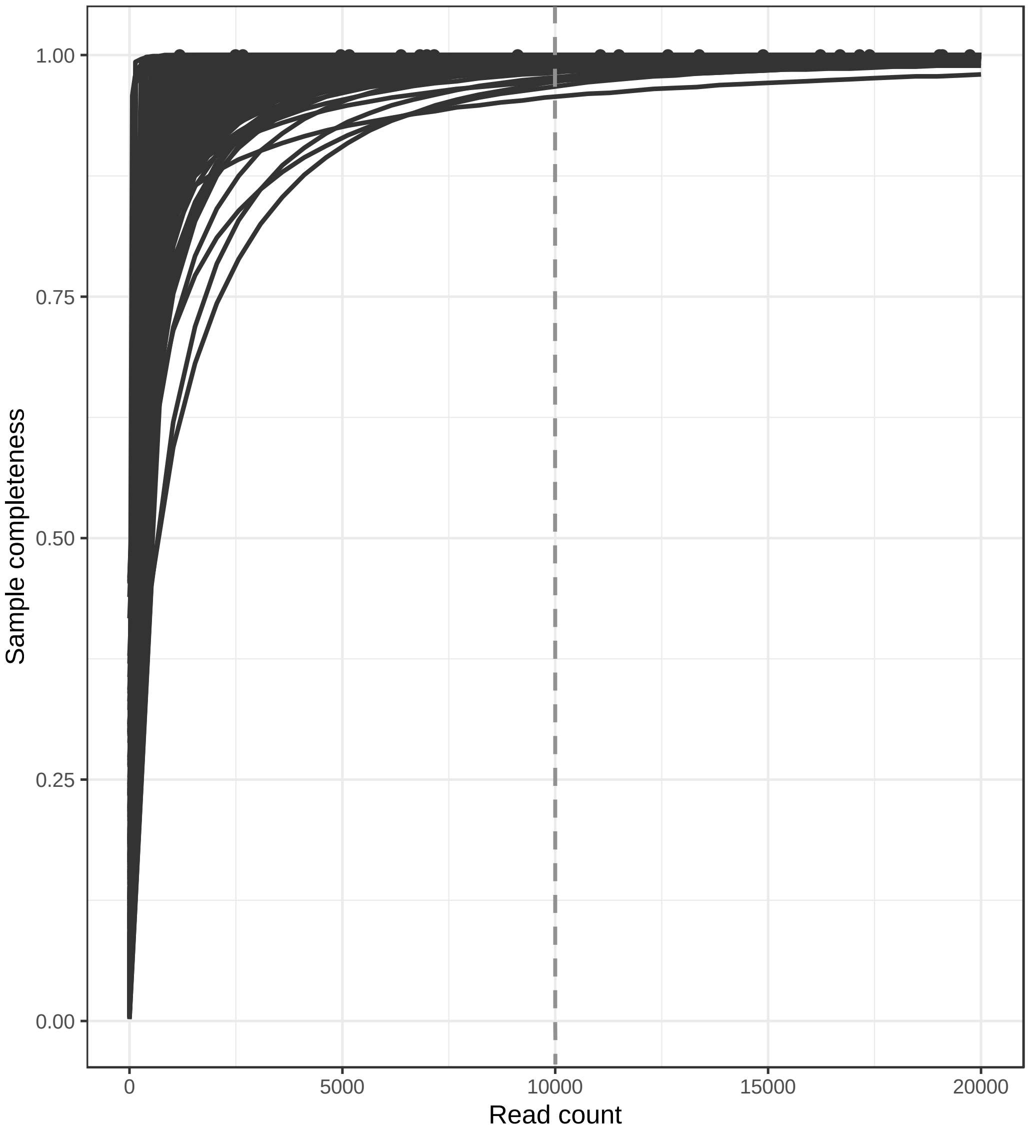

Figure S1. Sample completeness curves. Curves were generated using the R package iNEXT 2.0.20, with 50 bootstrap replicates per sample. The dashed line represents the number of reads used as a cut-off for retaining samples in downstream analysis (all samples with fewer than 10,000 reads were removed).

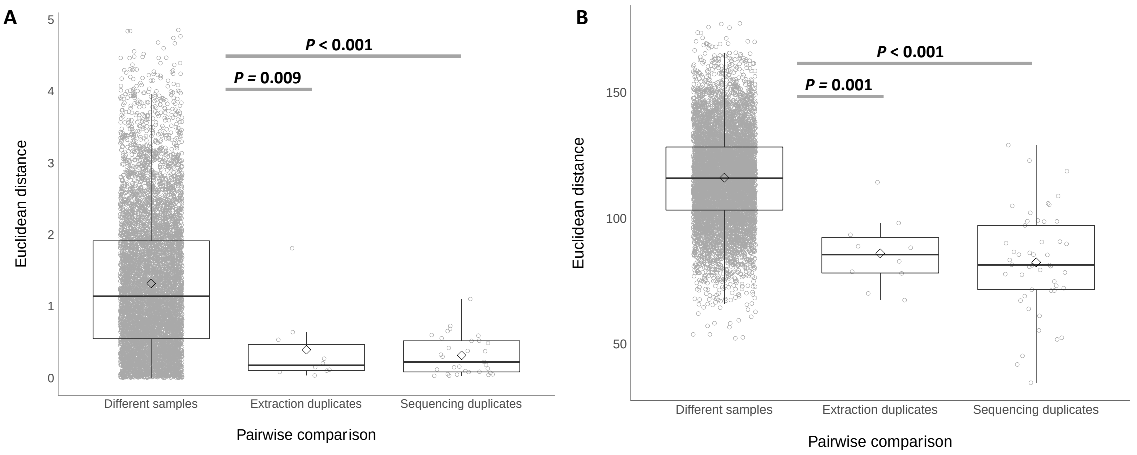

Figure S2. The similarity of **A)** alpha, and **B)** beta diversity measures across Seychelles warbler faecal samples. Pairwise Euclidean distances were calculated between samples where the same DNA had been sequenced twice (sequencing duplicates n = 49 samples after filtering), samples from which DNA had been extracted and sequenced twice (extraction duplicates, n =10 samples) and for pairs of different faecal samples (different samples, n = 59 samples in total). Alpha diversity was calculated as Shannon diversity and beta diversity was calculated using CLR-transformed ASV abundances. Boxes span the interquartile (25% - 75%) range. The median is marked by a horizontal line and the mean is marked by a diamond. Whiskers extend to 1.5 times the interquartile range. Significant differences are shown; P-values are derived from bootstrapped Dunn's multiple comparison tests.

Table S1. The results of post-hoc pairwise PERMANOVA analyses investigating differences in gut microbiome composition across A) age classes and B) sampling periods. Analyses for age class (A) were based on Euclidean distances calculated using CLR transformed Amplicon Sequencing Variant (ASV) abundances. FL = fledglings, OFL = old fledglings, SA = sub-adults, and A = adults. Both (i) CLR- and (ii) PhILR transformed abundances were analysed in post-hoc tests of sampling period (B). Significant differences in composition (P_{adj} < 0.05) are shown in bold. *P* values were adjusted for multiple testing using the Benjamini and Hochberg correction.

Pairwise comparison	*df*	*F*	P_{adj}
**A) Age class**			
A - SA	1	1.181	0.110
**A - FL**	**1**	**1.525**	**0.030**
A - OFL	1	1.169	0.110
SA - FL	1	1.242	0.090
SA - OFL	1	1.165	0.110
**OFL - FL**	**1**	**1.541**	**0.006**
**B) Sampling period**			
**i) CLR**			
**Major17 - Major 18**	**1**	**1.992**	**0.001**
**Major17 - Minor18**	**1**	**1.559**	**0.003**
**Major17 - Major19**	**1**	**3.115**	**0.001**
**Major17 – Minor20**	**1**	**5.021**	**0.001**
**Major17 - Minor19**	**1**	**3.741**	**0.001**
**Major18 - Minor18**	**1**	**1.623**	**0.008**
**Major18 - Major19**	**1**	**4.198**	**0.001**

**Major18 – Minor20**	**1**	**7.413**	**0.001**
**Major18 - Minor19**	**1**	**7.054**	**0.001**
**Minor18 - Major19**	**1**	**2.348**	**0.001**
**Minor18 – Minor20**	**1**	**2.720**	**0.001**
**Minor18 - Minor19**	**1**	**2.139**	**0.001**
**Major19 – Minor20**	**1**	**3.072**	**0.001**
**Major19 - Minor19**	**1**	**2.371**	**0.001**
**Minor20 – Minor19**	**1**	**1.400**	**0.020**
**ii) PhILR**			
**Major17 - Major 18**	**1**	**1.858**	**0.046**
Major17 - Minor18	1	1.337	0.175
**Major17 - Major19**	**1**	**2.087**	**0.023**
**Major17- Minor20**	**1**	**3.969**	**0.002**
**Major17 - Minor19**	**1**	**3.482**	**0.003**
**Major18 - Minor18**	**1**	**2.971**	**0.002**
**Major18 - Major19**	**1**	**6.219**	**0.002**
**Major18 – Minor20**	**1**	**7.800**	**0.002**
**Major18 - Minor19**	**1**	**12.769**	**0.002**
Minor18 - Major19	1	1.278	0.180
**Minor18 – Minor20**	**1**	**2.383**	**0.010**
Minor18 – Minor19	1	1.529	0.089
**Major19 – Minor20**	**1**	**3.945**	**0.002**
**Major19 – Minor19**	**1**	**3.465**	**0.002**
Minor20 – Minor19	1	1.742	0.050

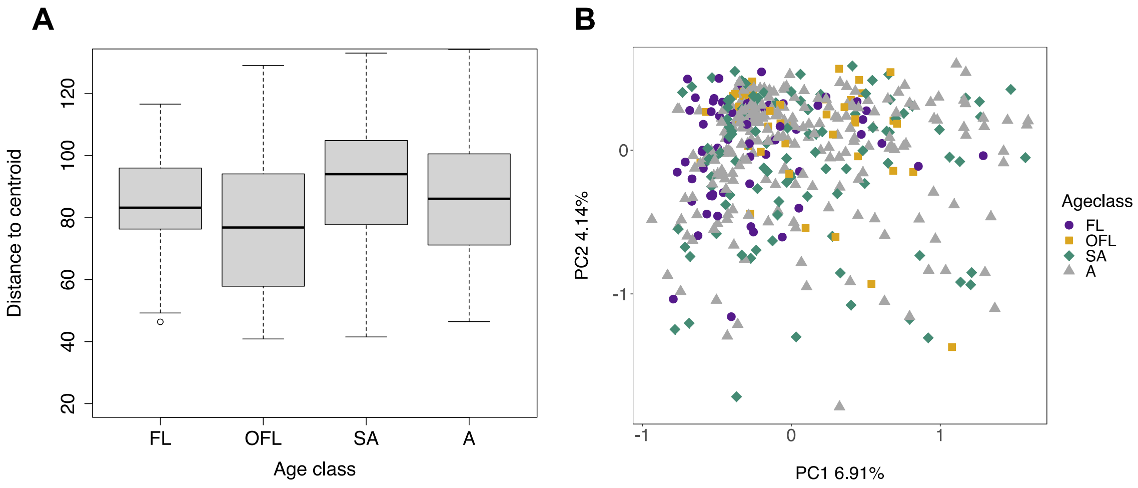

Figure S3. Differences in gut microbiome (GM) composition across age classes in the Seychelles warbler. **A)** Results of a betadisper analysis showing significant differences ($F_{3,446}$ = 6.062, P < 0.001) in GM variability (distance to centroid) across age classes. Boxes encompass the interquartile (25%-75%) range and the median is marked by a horizontal line. Whiskers extend to 1.5 times the interquartile range. **B)** A Principal Components Analysis (PCA) of Euclidean distances between the GM of individuals in different age classes. Euclidean distances are based on CLR-transformed abundances of amplicon sequencing variants (ASVs). Principal components one and two explain 6.91% and 4.14% of the variation in GM structure, respectively. Each point represents a single sample. N = 450 faecal samples from 309 individuals; 65 samples = FL (fledglings), 45 = OFL (old fledglings), 106 = SA (sub-adults), 234 = A (adults).

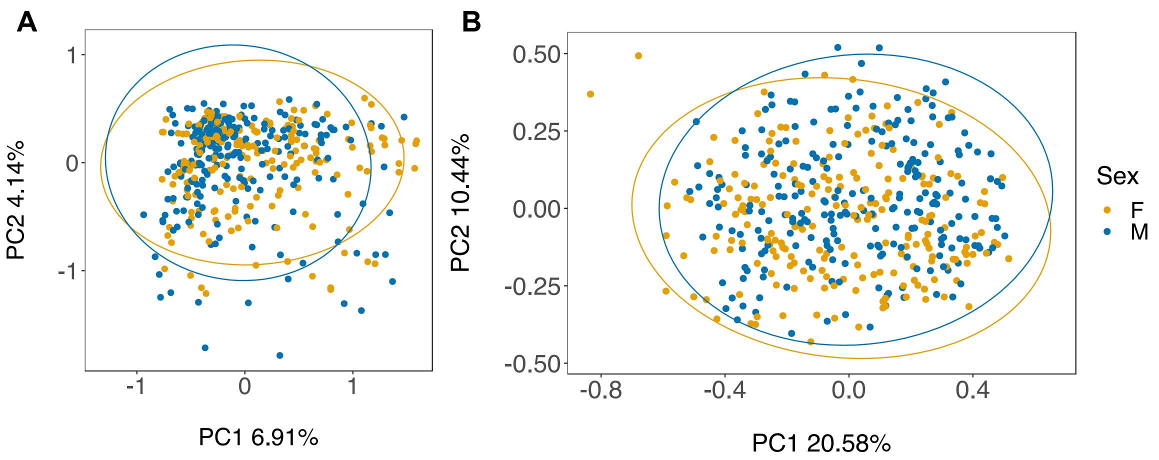

Figure S4. A Principal Components Analysis (PCA) of Euclidean distances between the gut microbiome (GM) of male (blue) and female (yellow) individuals. Euclidean distances are based on **A)** CLR- or **B)** PhILR- transformed abundances of amplicon sequencing variants (ASVs). Ellipses denote 95% confidence intervals. Principal components one and two explain 6.91% and 4.14% of the variation in GM structure, respectively. Each point represents a single sample. N = 450 faecal samples from 309 individuals; 213 samples = Female, 237 samples = Male.

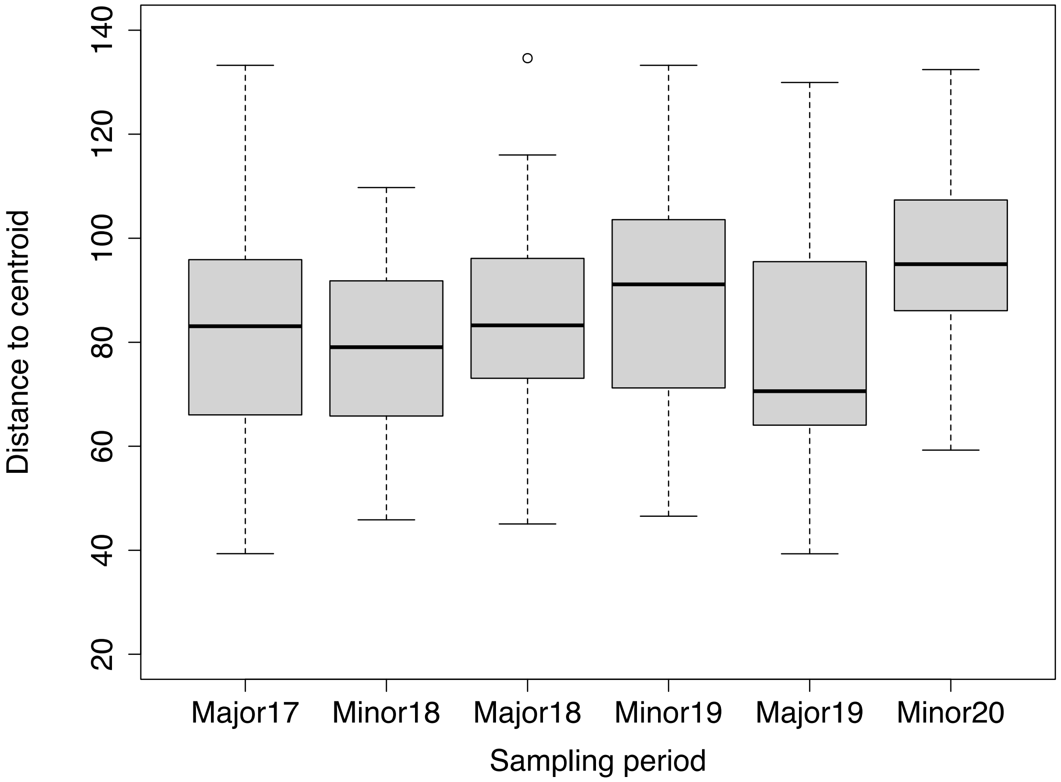

Figure S5. Results of a betadisper analysis showing differences in gut microbiome variability (distance to centroid) across sampling periods. Boxes encompass the interquartile (25%-75%) range and the median distance is marked by a horizontal line. Whiskers extend to 1.5 times the interquartile range.

Table S2. Linear Mixed Model analyses investigating the association between gut microbiome alpha diversity and body condition in the Seychelles warbler. Alpha diversity was measured as A) Shannon diversity, B) Chao1 richness and C) Faith's PD; these metrics were included in separate models and *P* values were adjusted for multiple hypothesis testing (P_{adj}) using the Benjamini and Hochberg method. Significant effects are shown in bold. Reference categories for categorical variables were as follows: adult (age class), female (sex) and major 2017 (field period). The marginal and conditional R^2 was 0.526 and 0.732 for A and B and 0.526 and 0.734 for model C, respectively.

Predictor	Estimate	SE	*df*	*t*	P_{adj}
**A) Shannon diversity**					
**Intercept**	**16.100**	**0.123**	**138.996**	**130.548**	**<0.001**
**Right tarsus**	**0.594**	**0.122**	**389.267**	**4.875**	**<0.001**
Shannon diversity	-0.056	0.074	371.787	-0.757	0.963
**Age class**					
**Fledgling**	**-0.939**	**0.115**	**393.914**	**-8.139**	**<0.001**
**Old fledgling**	**-0.554**	**0.129**	**391.887**	**-4.302**	**<0.001**
**Sub-adult**	**-0.612**	**0.094**	**397.720**	**-6.494**	**<0.001**
**Sex (Male)**	**0.805**	**0.126**	**342.016**	**6.368**	**<0.001**
Territory quality	-0.152	0.112	330.673	-1.355	0.176
**Time of day**	**0.238**	**0.077**	**377.753**	**3.098**	**0.002**
**Field period**					

**Minor 2018**	**-0.581**	**0.162**	**151.237**	**-3.591**	**<0.001**
**Major 2018**	**-0.428**	**0.145**	**165.966**	**-2.944**	**0.004**
**Minor 2019**	**-0.811**	**0.166**	**77.416**	**-4.898**	**<0.001**
Major 2019	-0.113	0.168	142.331	-0.673	0.545
**Minor 2020**	**-1.082**	**0.154**	**77.815**	**-7.018**	**<0.001**
**Random factors**	**425 samples**	**Variance**			
Bird ID	296 individuals	0.225			
Territory ID	101 territories	0.050			
Observer ID	12 observers	0.001			
**B) Chao1 Richness**					
**Intercept**	**16.102**	**0.124**	**143.741**	**129.839**	**<0.001**
**Right tarsus**	**0.594**	**0.122**	**388.744**	**4.872**	**<0.001**
Chao1 richness (log)	0.011	0.077	323.133	0.137	0.963
**Age class**					
**Fledgling**	**-0.939**	**0.116**	**393.589**	**-8.130**	**<0.001**
**Old fledgling**	**-0.539**	**0.129**	**393.318**	**-4.171**	**<0.001**
**Sub-adult**	**-0.615**	**0.094**	**397.264**	**-6.516**	**<0.001**
**Sex (Male)**	**0.811**	**0.126**	**341.830**	**6.421**	**<0.001**
Territory quality	-0.155	0.112	333.600	-1.378	0.176
**Time of day**	**0.237**	**0.077**	**377.828**	**3.078**	**0.002**

**Field period**					
**Minor 2018**	**-0.578**	**0.162**	**152.061**	**-3.570**	**<0.001**
**Major 2018**	**-0.432**	**0.146**	**167.526**	**-2.965**	**0.004**
**Minor 2019**	**-0.814**	**0.168**	**83.212**	**-4.850**	**<0.001**
Major 2019	-0.102	0.168	144.915	-0.607	0.545
**Minor 2020**	**-1.095**	**0.158**	**86.158**	**-6.909**	**<0.001**
**Random factors**	**425 samples**	**Variance**			
Bird ID	296 individuals	0.223			
Territory ID	101 territories	0.049			
Observer ID	12 observers	0.001			
**B) Faith's PD**					
**Intercept**	**16.100**	**0.124**	**141.619**	**130.119**	**<0.001**
**Right tarsus**	**0.593**	**0.122**	**388.616**	**4.869**	**<0.001**
Faith's PD	-0.004	0.075	322.626	-0.047	0.963
**Age class**					
**Fledgling**	**-0.939**	**0.116**	**393.717**	**-8.128**	**<0.001**
**Old fledgling**	**-0.542**	**0.129**	**393.120**	**-4.202**	**<0.001**
**Sub-adult**	**-0.614**	**0.094**	**397.237**	**-6.509**	**<0.001**
**Sex (Male)**	**0.810**	**0.126**	**341.880**	**6.414**	**<0.001**
Territory quality	-0.153	0.112	331.908	-1.368	0.176

**Time of day**	0.238	0.077	378.418	3.084	**0.002**
**Field period**					
**Minor 2018**	**-0.578**	**0.162**	**152.189**	**-3.564**	**<0.001**
**Major 2018**	**-0.430**	**0.146**	**166.769**	**-2.953**	**0.004**
**Minor 2019**	**-0.809**	**0.167**	**80.286**	**-4.851**	**<0.001**
Major 2019	-0.102	0.168	142.808	-0.608	0.545
**Minor 2020**	**-1.088**	**0.157**	**81.325**	**-6.939**	**<0.001**
**Random factors**	**425 samples**	**Variance**			
Bird ID	296 individuals	0.223			
Territory ID	101 territories	0.049			
Observer ID	12 observers	0.001			

Table S3. PERMANOVA analysis of gut microbiome distances and body condition in **A)** juvenile and **B)** adult Seychelles warblers. Euclidean distances were calculated based on either CLR or PhILR transformed Amplicon Sequencing Variant (ASV) abundances. Significant predictors (P < 0.05) are shown in bold. Analyses included 205 samples from juveniles (175 individuals), and 220 samples from adults (165 individuals), respectively. Bird ID was included as a blocking factor in analyses to control for repeated sampling.

**Predictor**	***df***	R^2		***F***		***P***	
		CLR	PhILR	CLR	PhILR	CLR	PhILR
**A) Juveniles**							
Age class	2	0.010	0.010	1.017	1.073	0.127	0.666
Sex	1	0.005	0.007	1.115	1.493	0.243	0.223
Territory quality	1	0.006	0.003	1.202	0.702	0.826	0.447
Sampling period	5	0.023	0.025	1.138	1.039	0.117	0.112
Body condition	1	0.008	0.010	1.600	1.998	0.262	0.229
**B) Adults**							
Sex	1	0.004	0.003	0.837	0.580	0.878	0.847
Territory quality	1	0.005	0.004	1.003	0.793	0.718	0.897
Sampling period	5	0.023	0.025	1.003	1.074	0.243	0.175
Body condition	1	0.004	0.003	0.974	0.666	0.788	0.538

Table S4. Generalised Linear Model investigating the association between gut microbiome alpha diversity and survival in the Seychelles warbler. The alpha diversity metrics A) Chao1 richness and B) Faith's PD and were included in separate models. Significant (P_{adj} <0.05) effects are shown in bold; *P* values were corrected for multiple hypothesis testing (using the Benjamini and Hochberg method) to control for the use of different alpha diversity metrics. Reference categories for categorical variables were as follows: adult (age class), female (sex) and 2017 (sample year). N = 264 individuals/samples were included in the analysis (226 individuals survived and 38 individuals died by the next breeding season).

Predictor	Estimate	SE	*z*	P_{adj}
**A) Chao1 Richness**				
**Intercept**	**1.517**	**0.611**	**2.485**	**0.013**
Chao1 richness	0.053	0.376	0.141	0.888
**Age class**				
Fledgling	-0.308	0.567	-0.543	0.626
**Old fledgling**	**-1.048**	**0.505**	**-2.073**	**0.042**
Sub-adult	0.921	0.581	1.583	0.123
Sex (Male)	-0.465	0.372	-1.248	0.230
Territory quality	1.248	0.698	1.789	0.075
Sample Year				
2018	0.604	0.725	0.832	0.408
2019	0.336	0.743	0.452	0.661
**B) Faith's PD**				
**Intercept**	**1.522**	**0.612**	**2.486**	**0.013**
Faith's PD	0.230	0.367	0.626	0.888

**Age class**				
Fledgling	-0.346	0.567	-0.611	0.626
**Old fledgling**	**-1.023**	**0.504**	**-2.031**	**0.042**
Sub-adult	0.894	0.580	1.542	0.123
Sex (Male)	-0.447	0.372	-1.200	0.230
Territory quality	1.256	0.699	1.796	0.075
Sample Year				
2018	0.615	0.727	0.846	0.408
2019	0.342	0.744	0.459	0.661

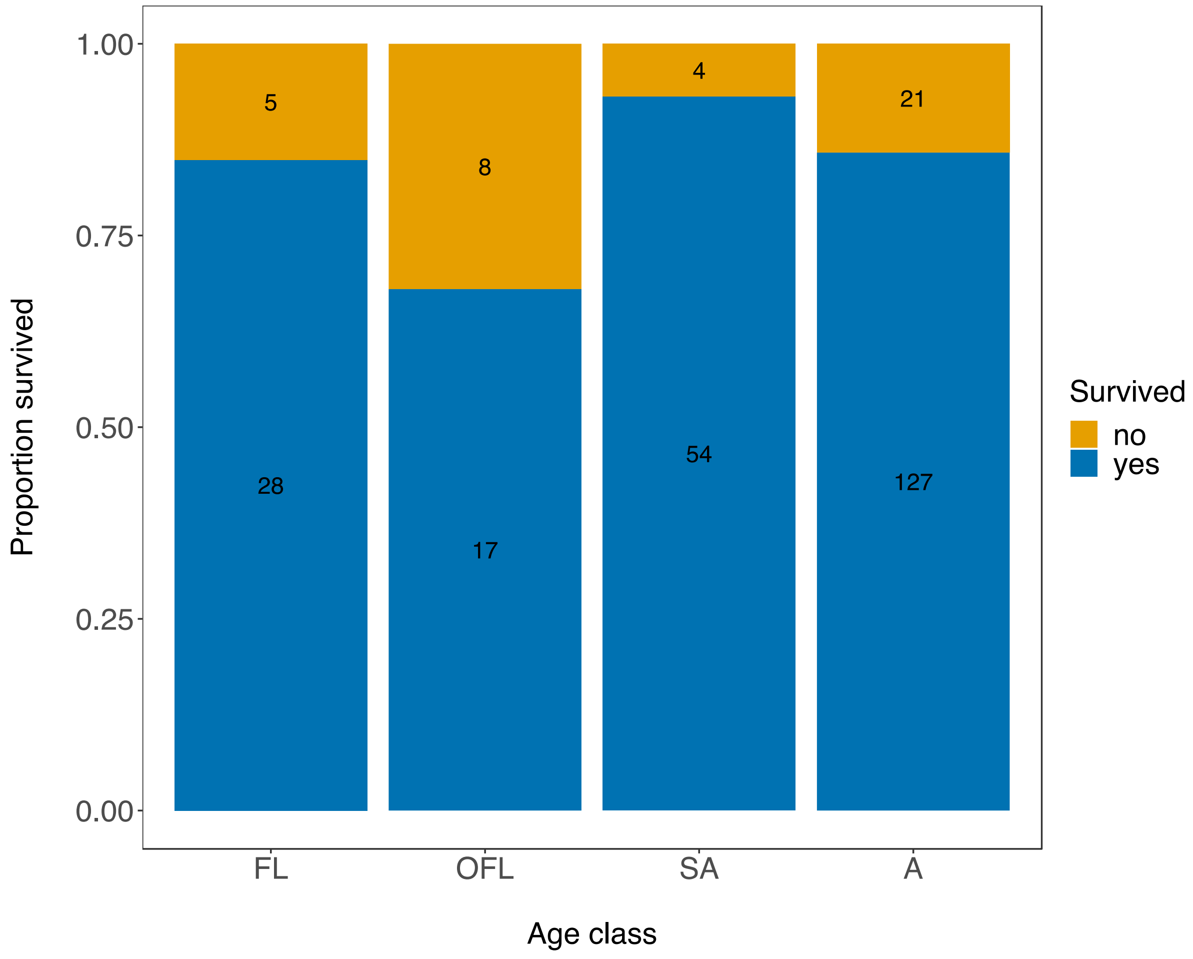

Figure S6. Survivorship across different bird age classes. FL = fledgling, OFL = old fledgling, SA = sub-adult, A = adult. Bars represent the proportion of birds sampled in each age class that were recorded as having survived (blue) or died (yellow) by the next breeding period. The raw numbers of individuals that survived or died are also presented. A total of 264 samples/individuals were included in survival analyses.

Table S5. Amplicon sequencing variants (ASVs) that were significantly, differentially abundant ($P_{adj} < 0.05$) in the gut microbiomes of adult Seychelles warbler individuals that survived versus those that died by the next breeding season. Effect sizes (log fold change) are shown with standard errors (SE). All *P*-values were adjusted with the Benjamini and Hochberg correction for multiple testing. A negative log fold change (white boxes) indicates that an ASV is more abundant in individuals that died by the next sampling period and a positive log fold change indicates a higher abundance in individuals that survived (grey boxes). Their taxonomic classification is shown to family level.

ASV-ID	Log fold change	SE	P_{adj}	Phylum	Order	Family
63eed2ab1eeb7f5411af7c7337b94b2e	-2.319	0.632	0.012	*Proteobacteria*	*Rhizobiales*	*Rhizobiaceae*
29183db0788cb488d53df46c835fee01	-1.859	0.410	0.003	*Actinobacteria*	*Solirubrobacterales*	*67-14*
4b6cf88795b029c8d12595ff6d43309f	-1.848	0.505	0.012	*Actinobacteria*	*Micrococcales*	*Microbacteriaceae*
7f3425f991fc7ef2d540a8471b692e20	-1.828	0.471	0.009	*Chloroflexi*	*Thermomicrobiales*	*JG30-KF-CM45*
ee181b79ce2c1fe91b44c3d6db56ae80	-1.762	0.486	0.012	*Chloroflexi*	*Thermomicrobiales*	*JG30-KF-CM45*
2ec50ce942d2d82b733c7c3478da8c05	-1.747	0.454	0.009	*Chloroflexi*	*Thermomicrobiales*	*JG30-KF-CM45*
57df434f08aafe972aa130f6c9a9a159	-1.734	0.467	0.012	*Actinobacteria*	*Corynebacteriales*	*Mycobacteriaceae*
f56c2a2c3898b9b05bbbc0a7887aa063	-1.705	0.531	0.030	*Chloroflexi*	*Thermomicrobiales*	*JG30-KF-CM45*
d1094f3f938cf893222a953ba8c484c8	-1.649	0.513	0.030	*Actinobacteria*	*Corynebacteriales*	*Mycobacteriaceae*
8651d51c63149243e6c122c5201098ae	-1.646	0.455	0.012	*Actinobacteria*	*Rubrobacterales*	*Rubrobacteriaceae*
76832c466c981b29d810b9f78c43a4f1	-1.628	0.503	0.030	*Chloroflexi*	*Thermomicrobiales*	*JG30-KF-CM45*

a99d6a1717c4468438578 ce3af464c9a	-1.568	0.465	0.022	*Actinobacteria*	*Solirubrobacterales*	*67-14*
97e6ad9b765f374a8755d d9db3bf9b18	-1.532	0.441	0.016	*Proteobacteria*	*Rhizobiales*	*Rhizobiaceae*
60abb0a1f07e3d8901d76 22ab322c2d0	-1.459	0.406	0.012	*Proteobacteria*	*Rhizobiales*	*Rhizobiaceae*
1a8f3c181db3d2137b4cb 3c4a76aaa4a	-1.459	0.407	0.012	*Proteobacteria*	*Rhizobiales*	*Beijerinckiaceae*
fcd7c36163689dc2885ef5 24555131f6	-1.443	0.396	0.012	*Actinobacteria*	*Propionibacteriales*	*Propionibacteriaceae*
906e2f1f081bd360b2bfd0 af92293de7	-1.441	0.467	0.041	*Proteobacteria*	*Rhizobiales*	*Rhizobiaceae*
5353bc8666c2ab14cadc1 7c42f7e6a03	-1.372	0.388	0.014	*Planctomycetes*	*Gemmatales*	*Gemmataceae*
860cddf8de6c6cfd0cd039 842cac77f9	-1.319	0.403	0.029	*Proteobacteria*	*Rhodobacterales*	*Rhodobacteraceae*
8d8e78b82245ed1345050 fe24c39f44b	-1.273	0.417	0.044	*Actinobacteria*	*Micrococcales*	*Micrococcaceae*
d421607669c05165abb7e 88b1e4304f0	-1.062	0.326	0.030	*Actinobacteria*	*Corynebacteriales*	*Mycobacteriaceae*
1e6dfa86ab0f479a5729c5 e0cd2ba7fb	-1.031	0.322	0.030	*Proteobacteria*	*Rhizobiales*	*Rhizobiaceae*
93cc16b5e1b72b9198198 249c164f930	0.806	0.255	0.033	*Firmicutes*	*Clostridiales*	*Family XIII*

449f566731e25ffaf4dedc35f07cb111	0.834	0.208	0.007	*Proteobacteria*	*Desulfovibrionales*	*Desulfovibrionaceae*
404e239a8ed1408bdf6ce803ac7321b3	1.048	0.251	0.005	*Proteobacteria*	*Enterobacteriales*	*Enterobacteriaceae*
203c07bb1ce9de25e17d75b18de74c56	1.376	0.336	0.006	*Proteobacteria*	*Rhizobiales*	*Rhizobiaceae*
1bc0480cbd20c56d1191c2987dc5f03e	1.428	0.408	0.015	*Proteobacteria*	*Rhodospirillales*	*Rhodospirillaceae*
dfc53c1ba6d9b7a481b0a44d7f2cce11	1.529	0.348	0.003	*Proteobacteria*	*Rhizobiales*	*Rhizobiaceae*
